# Tocilizumab-associated oral ulcerations: a case report and scoping review

**DOI:** 10.3389/fmed.2026.1905880

**Published:** 2026-09-16

**Authors:** Suad Aljohani, Ebtihaj T. Nafea

**Affiliations:** 1 Department of Oral Diagnostic Sciences, Faculty of Dentistry, King Abdulaziz University, Jeddah, Saudi Arabia; 2 Department of Preventive Dental Sciences, Dental College, Taibah University, Madinah, Saudi Arabia

**Keywords:** oral-systemic interactions, adverse drug reactions, interleukin-6, mouth ulcer, rheumatoid arthritis, tocilizumab

## Abstract

**Objectives:**

Tocilizumab (TCZ) is an interleukin-6 receptor inhibitor used in the treatment of rheumatoid arthritis and other inflammatory disorders. Oral ulcerative events have been described as an infrequently reported and potentially underrecognized effect of TCZ, but their clinical characteristics, frequency, and relationship to treatment remain incompletely characterized. This study aimed to map and characterize the available clinical evidence on oral ulcerative events reported during TCZ treatment and to present a clinical case.

**Materials and methods:**

A scoping review was conducted and reported in accordance with PRISMA-ScR guidelines. Peer-reviewed studies reporting oral ulceration, stomatitis, aphthous ulceration, or related oral mucosal events in adults receiving TCZ were included. A clinical case of recurrent oral ulceration temporally associated with TCZ therapy was also described.

**Results:**

Seventeen studies were included, comprising six clinical trials, five observational studies, and six case-report publications. Oral events were variably reported as oral or mouth ulceration, stomatitis, aphthous ulceration, or mucosal ulceration. Detailed information regarding lesion morphology, anatomical distribution, time to onset, duration, and resolution was frequently unavailable, particularly in clinical trials and observational studies. Reported events represented distinct clinical patterns, including newly occurring treatment-emergent events, recurrence following rechallenge, and persistence, lack of therapeutic response, or worsening of pre-existing disease-related ulceration. In the present case, recurrent painful oral ulcerations appeared approximately 24 h after the first TCZ injection and recurred following subsequent doses. Their frequency and severity gradually decreased following multidisciplinary management and subsequent discontinuation of TCZ, with complete resolution reported at the latest follow-up, nine months after TCZ withdrawal.

**Conclusion:**

Oral ulcerative events are infrequently reported during TCZ therapy and may be underrecognized; however, the available evidence is heterogeneous and does not establish a uniform clinical phenotype or a definitive causal relationship. Pre-existing disease-related ulceration should be distinguished from newly occurring treatment-emergent events. Careful assessment of temporal relationships, underlying disease, concomitant therapies, and alternative etiologies is important when evaluating oral ulceration in patients receiving TCZ.

## Introduction

Tocilizumab (TCZ) is a humanized monoclonal antibody targeting the interleukin-6 (IL-6) receptor and is approved for the treatment of rheumatoid arthritis and several other autoimmune and inflammatory disorders. By inhibiting IL-6 signalling, TCZ exerts potent anti-inflammatory effects and effectively reduces disease activity. However, this therapeutic mechanism may also compromise host immune defence and disrupt mucosal integrity. This is attributable to the pleiotropic nature of IL-6, which plays a central role not only in immune regulation but also in mucosal homeostasis, epithelial regeneration, and tissue repair (1–3).

Although TCZ is generally well tolerated, a range of adverse effects has been reported, including infections, hepatotoxicity, and hematologic abnormalities such as neutropenia (3, 4). Less commonly recognized are mucocutaneous adverse effects, including oral ulcerations, which remain underappreciated in clinical practice despite their potential impact on oral function and quality of life. These lesions may present as painful erosions or ulcers of the oral mucosa and can interfere with nutritional intake and overall well-being. The pathophysiological mechanisms underlying TCZ-associated oral ulcerations are not fully understood but are thought to involve the immunosuppressive effects of IL-6 inhibition, leading to impaired mucosal barrier integrity and diminished local immune defense (5, 6). Furthermore, TCZ-associated neutropenia may increase susceptibility to mucosal injury by reducing immune surveillance and host defense (4).

Clinical evidence supporting the association between TCZ and oral ulcerations remains limited but is gradually expanding. Case reports have documented recurrent aphthous-like ulcerations temporally associated with TCZ administration, with resolution upon drug discontinuation and recurrence following rechallenge (7, 8). In patients with Behçet's disease, findings are heterogeneous, with some studies reporting persistence or worsening of mucosal lesions despite systemic improvement (9, 10).

Beyond individual case reports, broader analyses indicate that mucocutaneous outcomes associated with TCZ are heterogeneous. A systematic review by Akiyama et al. demonstrated that while some patients with Behçet's disease experienced improvement in mucosal symptoms, others developed worsening oral and genital ulcerations during therapy (11). Additionally, reports of intestinal mucosal ulceration in patients receiving TCZ further support the hypothesis that IL-6 receptor blockade may impair mucosal repair (12–14). Given these potential risks, careful monitoring for oral discomfort or mucosal lesions is warranted in patients receiving TCZ, particularly in those with predisposing factors such as concurrent immunosuppressive therapy or poor oral hygiene.

Despite sporadic reports, oral ulcerations associated with TCZ remain poorly characterized and are not consistently emphasized in drug safety profiles. The immunomodulatory effects of IL-6 inhibition, combined with TCZ-induced neutropenia, may plausibly contribute to impaired mucosal immune defense and delayed epithelial healing. To date, no systematic review has comprehensively examined the occurrence, clinical characteristics, and underlying mechanisms of TCZ-associated oral ulcerations. Collectively, existing evidence suggests that TCZ may predispose patients to or exacerbate mucosal ulceration potentially through disruption of IL-6–mediated epithelial healing, modulation of T-cell responses, or alterations in the mucosal microbiota (8).

Recognition of this potential oral-systemic interaction is clinically important, as oral ulcerations in patients receiving biologic therapies are often misattributed to underlying disease activity, infection, or nutritional deficiencies. Accordingly, the differential diagnosis should include infectious etiologies, nutritional deficiencies, and the effects of concomitant medications. Management is primarily supportive and includes pain control, topical therapies, and optimization of oral hygiene.

Herein, we report a case of recurrent oral ulcerations temporally associated with TCZ therapy and present a scoping review of the available clinical evidence. The objective of this review was to map and characterize oral ulcerative events reported during TCZ treatment, including their reported frequency, clinical context, temporal relationship to treatment, and outcomes across different study designs and patient populations. Given the heterogeneous and predominantly descriptive nature of the available evidence, a scoping review was considered appropriate to characterize the existing literature and identify gaps in current knowledge rather than to establish causality or estimate treatment effects.

## Methods (scoping review)

A scoping review was conducted to explore the association between TCZ therapy and oral ulcerations. The review was performed in accordance with established scoping review frameworks and is reported following the Preferred Reporting Items for Systematic Reviews and Meta-Analyses extension for Scoping Reviews (PRISMA-ScR) guidelines (15). This approach was selected to enable systematic mapping of the heterogeneous and predominantly descriptive literature and to identify patterns in reported oral manifestations, clinical context, management, and gaps in the available evidence (16).

### Research question and objectives

The primary research question was:

What are the reported clinical characteristics, temporal patterns, and management strategies of oral ulcerative events reported during TCZ treatment?

Given the descriptive nature of the available evidence, a formal comparator was not applicable.

### Search strategy

A comprehensive literature search was conducted to identify reports of oral ulcerative events during TCZ therapy. PubMed/MEDLINE, Embase, and Web of Science Core Collection were searched from database inception through August 11, 2026. ScienceDirect was searched as a supplementary publisher-platform source, and the Cochrane Central Register of Controlled Trials (CENTRAL) was searched on August 13, 2026. No date restrictions were applied. Search strategies combined controlled vocabulary, where available, with free-text terms related to TCZ and oral ulcerative events and were adapted to the indexing system and syntax of each database.

#### PubMed/MEDLINE

The following search strategy was used:

#1 (“tocilizumab"[Supplementary Concept] OR tocilizumab[tiab] OR Actemra[tiab] OR RoActemra[tiab] OR atlizumab[tiab])
#2 (“Oral Ulcer"[MeSH Terms] OR “Stomatitis"[MeSH Terms] OR “Stomatitis, Aphthous"[MeSH Terms] OR “Mucositis"[MeSH Terms] OR “oral ulcer*"[tiab] OR “mouth ulcer*"[tiab] OR “oral mucosal ulcer*"[tiab] OR “mucosal ulcer*"[tiab] OR aphth*[tiab] OR stomatitis[tiab] OR mucositis[tiab] OR “oral erosion*"[tiab] OR “oral mucosal lesion*"[tiab] OR “mouth sore*"[tiab])
#3 #1 AND #2

#### Embase

The following search strategy was used:

#1 ‘tocilizumab'/de OR tocilizumab,ab OR actemra,ab OR roactemra,ab OR atlizumab,ab
#2 ‘oral ulcer'/de OR ‘aphthous stomatitis’/de OR ‘oral ulcer*’,ab OR ‘mouth ulcer*’,ab OR ‘aphthous ulcer*’,ab OR ‘aphthous stomatitis’,ab OR ‘oral mucositis’,ab OR ‘oral mucosal ulcer*’,ab
#3 #1 AND #2

#### Web of science core collection

A Topic search was performed using the following concepts:

#1 tocilizumab OR actemra OR roactemra OR atlizumab
#2 “oral ulcer*” OR “mouth ulcer*” OR “aphthous ulcer*” OR “aphthous stomatitis” OR “oral mucositis” OR “oral mucosal ulcer*” OR stomatitis OR aphth*
#3 #1 AND #2

#### Sciencedirect

ScienceDirect was searched as a supplementary source using the following combination of terms:

tocilizumab AND (“oral ulcer” OR “oral ulcers” OR “aphthous ulcer” OR “aphthous stomatitis” OR “oral mucositis” OR “oral mucosal ulcer”)

#### Cochrane central register of controlled trials (CENTRAL)

CENTRAL, accessed through the Cochrane Library, was searched using the following free-text strategy:

#1 tocilizumab OR Actemra OR RoActemra OR atlizumab
#2 oral ulcer OR aphthous ulcer OR stomatitis OR mucositis
#3 #1 AND #2

#### Reference and citation searching

The electronic database searches were supplemented by backward citation searching of the reference lists of included studies.

### Eligibility criteria

#### Inclusion criteria

- Studies reporting oral ulcerations associated with TCZ therapy, including oral mucositis, aphthous ulcers, or stomatitis
- Clinical trials, observational studies, case reports or case series, and correspondence/letters containing primary clinical data
- Adult patients receiving TCZ
- TCZ administered via any route (e.g., intravenous or subcutaneous) and at any dosage
- Full-text articles published in peer-reviewed journals

#### Exclusion criteria

- Animal, *in vitro*, or preclinical studies
- Studies in which oral ulcerations were clearly attributable to alternative causes (e.g., infections, malignancy, or other medications)
- Review articles, conference abstracts without full text, editorials, and commentaries lacking primary clinical data
- Duplicate publications or studies reporting overlapping datasets
- Study Selection

Two independent reviewers (SA, EN) screened titles and abstracts for eligibility. Full-text articles of potentially relevant studies were then assessed independently. Any discrepancies were resolved through discussion and consensus.

### Data extraction

Data were extracted using a standardized data collection form, including:

- Study characteristics
- Patient demographics
- Underlying disease indication
- TCZ dose, route, and duration
- Description and timing of oral ulcerations
- Management strategies and clinical outcomes

## Results

### Study selection

The database searches identified 542 records, including 33 from PubMed, 345 from Embase, 33 from Web of Science, 124 from ScienceDirect, and 7 from Cochrane CENTRAL. After removal of 89 duplicate records, 453 records underwent title and abstract screening, of which 363 were excluded as clearly irrelevant. Ninety reports were sought for retrieval, and all were obtained and assessed for eligibility. Of these, 76 reports were excluded because they did not meet the inclusion criteria (*n* = 39), had an ineligible study design (*n* = 11), or provided insufficient relevant data or no eligible oral-ulcer outcome (*n* = 26). This resulted in 14 eligible studies identified through database searching. An additional three eligible studies were identified through citation searching, all of which were retrieved and assessed, with no exclusions. Thus, 17 studies were included in the scoping review, represented by 17 reports (Figure 1).

**Figure 1:**
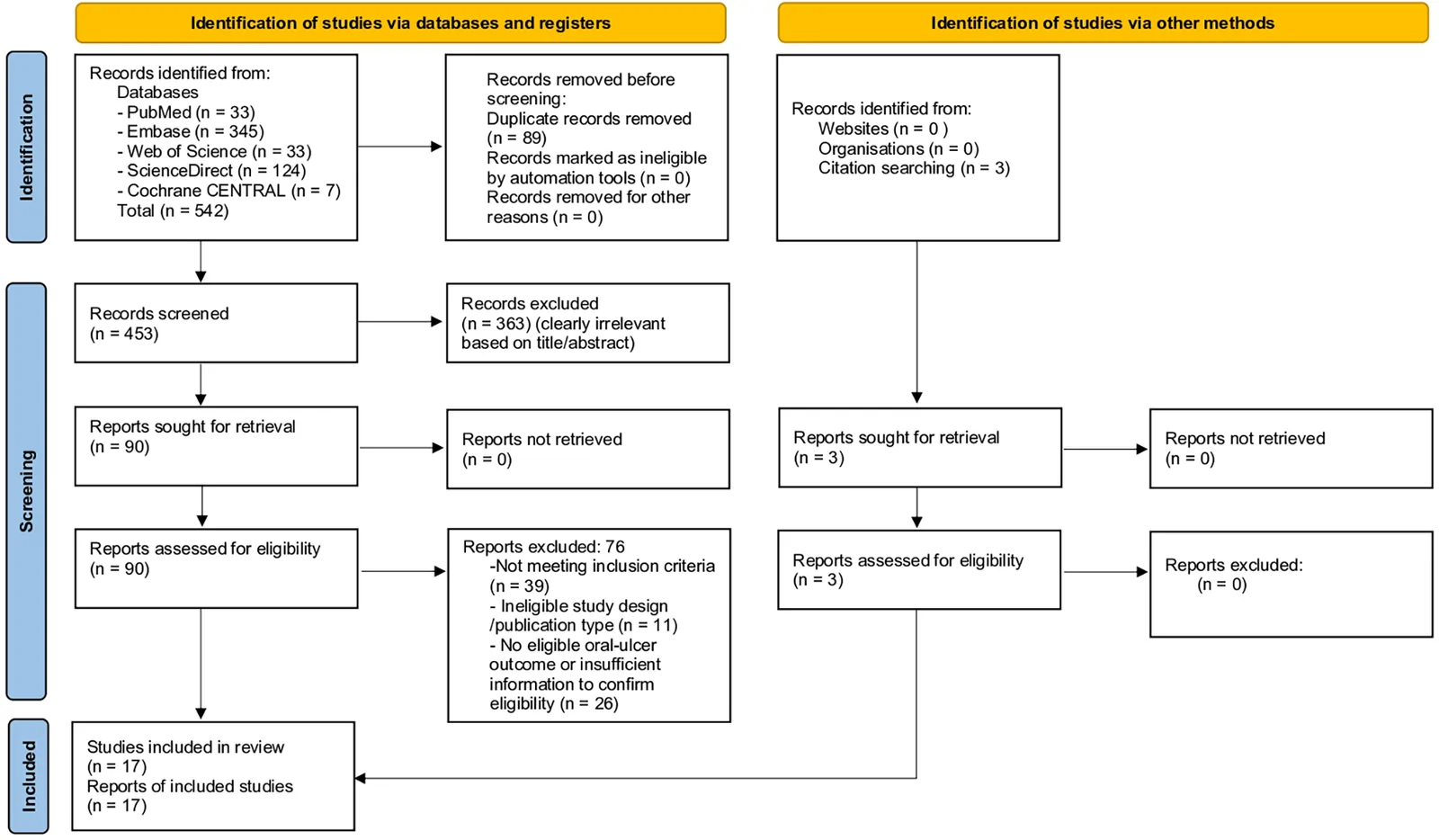
PRISMA flow diagram illustrating the study selection process.

### Characteristics of included studies

The 17 included publications comprised six clinical trials, five observational studies, and six case-report publications. Study populations included patients with rheumatoid arthritis, Behçet's disease, adult-onset Still disease, relapsing polychondritis, MAGIC syndrome, other refractory autoimmune diseases, and healthy adult volunteers. TCZ was administered intravenously or subcutaneously, either as monotherapy or in combination with conventional immunosuppressive therapies. The characteristics of the included studies and reported oral outcomes are summarized in Table 1.

**Table 1:** Characteristics of studies reporting oral ulcerative events during tocilizumab therapy.

Study	Design	Population	Underlying condition	TCZ regimen	Route	Onset of oral event	Oral outcome
Nishimoto et al. (SAMURAI) (17)	Randomized, x-ray-reader-blinded controlled trial	306 randomized; 302 treated (TCZ *n* = 157; conventional DMARDs *n* = 145)	Active RA with inadequate response to DMARDs	TCZ monotherapy 8 mg/kg every 4 weeks for 52 weeks vs. conventional DMARDs	IV	NR	Stomatitis: 9/157 (5.7%) with TCZ vs. 13/145 (9.0%) with conventional DMARDs; resolution NR.
Nishimoto et al. (SATORI) (18)	Randomized, double-blind controlled trial	127 randomized; 125 treated (TCZ *n* = 61; control *n* = 64)	Active RA with inadequate response to low-dose MTX	TCZ 8 mg/kg every 4 weeks + MTX placebo for 24 weeks vs. placebo + MTX 8 mg/week	IV	NR	Stomatitis: 7/61 (11.5%) with TCZ vs. 0/64 control; resolution NR.
Genovese et al. (TOWARD) (4)	Phase III randomized, double-blind, placebo-controlled trial	1,220 randomized; safety population (TCZ *n* = 802, placebo *n* = 414)	Active RA with inadequate response to conventional DMARDs	TCZ 8 mg/kg every 4 weeks + stable DMARDs vs. placebo + stable DMARDs	IV	NR	Mouth ulceration: 2.0% with TCZ vs. 1.0% placebo; stomatitis: 1.0% vs. 0%; onset/resolution NR.
Smolen et al. (OPTION) (19)	Phase III randomized, double-blind, placebo-controlled trial	622 patients were included (TCZ 4 mg/kg *n* = 212; TCZ 8 mg/kg *n* = 206; placebo *n* = 204).	RA with inadequate response to MTX	TCZ 4 or 8 mg/kg every 4 weeks + stable MTX 10–25 mg/week vs. placebo + MTX	IV	NR	Mouth ulcers: 4/212 (2.0%) with TCZ 4 mg/kg; 5/206 (2.0%) with TCZ 8 mg/kg; 1/204 (0.5%) with placebo; resolution NR.
Qian et al. (20)	Phase I randomized, double-blind, parallel-controlled, single-dose trial	80 healthy males randomized; 77 treated/completed (HS628 *n* = 40; reference TCZ *n* = 37); age 18–40 years	Healthy volunteers	Single 4 mg/kg dose of HS628 or reference TCZ	IV	NR	Oral ulcer: 10/40 (25.0%) with HS628 vs. 9/37 (24.3%) with reference TCZ; oral-ulcer-specific resolution NR.
Schwabe et al. (21)	Randomized, double-blind, parallel-group, single-dose trial	685 healthy adults, age 18–55 years; mean age 27 ± 7.6 years; 52% male	Healthy volunteers	Single 162 mg dose: MSB11456 (*n* = 231), US-TCZ (*n* = 229), or EU-TCZ (*n* = 225)	SC	NR	Mouth ulceration: 23/685 (3.4%) overall; 7/231 (3.0%) MSB11456, 13/229 (5.7%) US-TCZ, 3/225 (1.3%) EU-TCZ; 1.9% considered treatment-related; resolution NR.
Kamal and Naeem (22)	Cross-sectional study	84 patients; mean age 34.44 ± 16.38 years (18–60); 18 M/66 F	RA refractory to conventional DMARDs	TCZ 4–8 mg/kg every 4 weeks with MTX/other DMARDs + low-dose prednisolone	IV	NR; late AEs monitored for 3 months	Oral ulcers: 1/84 (1.2%); resolution NR.
Michaud et al. (23)	Retrospective multicentre study	57 patients; mean age 56 ± 16.4 years (23–80); 20 M/37 F	Various refractory autoimmune diseases	8 mg/kg every 4 weeks in IV cases; MTX co-administered in 4 (7%)	Predominantly IV (96%)	NR	Oral ulcers: 1/57 (1.8%); resolution NR.
Pers et al. (24)	Multicentre retrospective cohort study	222 RA patients; median age 56 years (IQR 47–66); 183 F (82.4%); 61 patients ≥65 years	RA	TCZ every 4 weeks; usual dose 8 mg/kg (median 8, range 4–8 mg/kg); dose adaptation allowed	IV	NR	Oral ulceration: 3/222 (1.4%); one disabling case with concomitant MTX; resolution NR.
Wang et al. (25)	Observational study	28 patients with refractory AOSD; 12 M/16 F	Adult-onset Still disease	TCZ 8 mg/kg every 4 weeks + MTX 12.5 mg/week; TCZ extended to every 8 weeks after 6-month remission	IV	NR	Oral ulcers: 4/28 (14.3%); self-limited improvement; exact timing NR.
Atienza-Mateo et al. (26)	Multicentre observational study	16 patients (10 M/6 F); mean age 36.5 ± 18.2 years	BD with refractory ocular and/or neurological involvement	8 mg/kg every 4 weeks (*n* = 13) or 162 mg weekly (*n* = 3); monotherapy or combined with conventional immunosuppressants	IV (13/16); SC (3/16)	Oral/genital ulcers were already present at TCZ initiation in 10/16 patients; individual onset time NR	Oral/genital ulcers improved in only 2/10 (20%); 8/10 showed no improvement/persistence; TCZ was permanently withdrawn in one patient because of persistent oral ulcers. Not *de novo* TCZ-associated oral ulceration. Not *de novo* TCZ-associated oral ulceration.
Tada et al. (27)	Case report	43-year-old woman	RA; previously treated with MTX	TCZ 8 mg/kg; at least 2 infusions; dosing interval NR	IV	20 days after 1st infusion	Stomatitis on the tongue developed 20 days after the first TCZ infusion, with concurrent cutaneous eruptions; TCZ was continued initially. Oral-event-specific resolution NR.
Samimi et al. (7)	Case report (2 patients)	2 females; aged 29 and 63-years	Case 1: ankylosing spondylitis; Case 2: RA	8 mg/kg monthly in both patients	IV	Case 1: 7 days after the second infusion; Case 2: 7 days after the first infusion.	Aphthous-like oral ulcers; resolved 6–7 weeks after TCZ withdrawal and recurred within ∼10 days after rechallenge in both patients. Colchicine was used in one case, with less severe recurrent episodes.
Vitiello et al. (8)	Case report	46-year-old male	Relapsing polychondritis	TCZ 8 mg/kg every 4 weeks	IV	After 2nd infusion	Ulcers persisted during TCZ; after the 13th infusion, peripheral arthritis also developed. TCZ was discontinued because of persistent oral ulceration and peripheral arthritis; oral ulcers progressively resolved within 15 days after withdrawal.
Cantarini et al., 2015 (10)	Case report	43-year-old female	BD with pre-existing recurrent oral aphthosis	TCZ 8 mg/kg/dose, with concomitant high-dose oral prednisone	IV	Pre-existing oral ulcers worsened 2 weeks after the first TCZ infusion; further mucocutaneous worsening occurred after the second infusion	Worsening of pre-existing Oral ulcers; TCZ discontinued; total recovery after switch to golimumab with colchicine and methylprednisolone. Not *de novo* TCZ-associated oral ulceration.
Diamantopoulos and Hatemi (28)	Case report	2 females, aged 55 and 26 years	BD with pre-existing mouth/genital ulcers	Case 1: 8 mg/kg; 1 infusion reported, with AZA 150 mg. Case 2: 8 mg/kg monthly × 3 infusions	IV	Case 1: worsening after 1st infusion; Case 2: timing NR	Case 1: Worsening of pre-existing oral ulcers after first TCZ infusion; TCZ discontinued after mucocutaneous worsening. Case 2: Oral-specific outcome NR; TCZ discontinued after 3 infusions because of lack of efficacy for mucocutaneous disease. Not *de novo* TCZ-associated oral ulceration.
Terreaux et al. (9)	Case report	37-year-old woman	MAGIC syndrome with pre-existing recurrent aphthous mouth ulcers and chondritis	TCZ; dose/schedule NR	NR	Pre-existing mouth ulcers; recurrent debilitating episodes after 4 months of TCZ	Persistence/recurrence of pre-existing mouth ulcers with chondritis and pseudofolliculitis despite TCZ; TCZ withdrawn for treatment failure; disease controlled after switch to infliximab. Not *de novo* TCZ-associated ulceration.

Where a detail was not explicitly reported in the accessible primary source, it is recorded as NR rather than inferred. For Behçet's disease reports, persistence or worsening of pre-existing mucocutaneous ulcers is distinguished from *de novo* treatment-emergent oral ulceration.

AE, adverse event; AZA, azathioprine; BD, Behçet's disease; DMARD, disease-modifying antirheumatic drug; EU, European Union; F, female; IQR, interquartile range; IV, intravenous; M, male; MAGIC, mouth and genital ulcers with inflamed cartilage; MTX, methotrexate; NR, not reported; RA, rheumatoid arthritis; RCT, randomized controlled trial; SC, subcutaneous; TCZ, tocilizumab; US, United State.

#### Clinical trials

Six clinical trials reported oral ulcerative events during TCZ exposure, including four randomized trials in patients with RA (4, 17–19), and two single-dose studies in healthy adults (20, 21). Reported events included stomatitis, mouth ulceration, and oral ulceration, with substantial variation in reported frequency across studies. Among the RA trials, oral events were reported more frequently in TCZ-treated groups than in comparator groups in three studies, whereas SAMURAI reported a lower frequency with TCZ than with conventional DMARDs. The healthy-volunteer studies also documented oral ulceration following exposure to reference TCZ or TCZ biosimilars; however, these studies were primarily designed to evaluate pharmacokinetics, comparability, and safety rather than oral adverse events. The characteristics and reported oral outcomes of these trials are summarized in Table 1.

#### Observational studies

Five observational studies reported oral ulcerative outcomes during TCZ treatment (22–26). Newly reported oral ulcers were generally infrequent, although a higher proportion was reported in the small Adult-onset Still disease cohort (25).

#### Case reports

Six case-report publications provided more detailed clinical and temporal information. Newly occurring oral ulcerative events during TCZ exposure were reported by Tada et al., Samimi et al., and Vitiello et al. (7, 8, 27). In the report by Samimi et al., oral ulcers resolved after TCZ withdrawal and recurred following rechallenge. In contrast, the reports by Cantarini et al., Diamantopoulos and Hatemi, and Terreaux et al. involved pre-existing disease-related mucocutaneous ulceration that persisted, showed limited therapeutic response, or worsened during TCZ treatment (9, 10, 28).

### Reported characteristics of oral events

Across the included studies, oral manifestations were variably reported as oral ulceration, mouth ulceration, stomatitis, aphthous ulceration, or mucosal ulceration. Information regarding lesion morphology, anatomical distribution, exact time to onset, duration, and resolution was frequently limited, particularly in clinical trials and observational studies, whereas more detailed descriptions were available in a limited number of case reports.

Based on the available temporal and clinical information, the reported events reflected several distinct patterns, including newly occurring treatment-emergent oral events, recurrence following rechallenge, persistence of pre-existing disease-related ulceration, lack of therapeutic response, and worsening or paradoxical flare of pre-existing mucocutaneous manifestations. Reports involving BD and MAGIC syndrome predominantly described persistence, inadequate response, or worsening of ulceration that predated TCZ treatment and were therefore distinguished from newly occurring oral events during TCZ exposure.

## Case report

The case was prepared in accordance with the CARE reporting guideline (29), with the clinical course presented chronologically.

A 53-year-old woman was referred to the Oral Medicine clinic for evaluation of painful, recurrent oral ulcerations. Her medical history was significant for rheumatoid arthritis, diagnosed 9 years ago, osteoporosis, hypertension, and diabetes mellitus. She was a nonsmoker and reported no history of recurrent oral ulceration before initiation of TCZ. No relevant gastrointestinal or other systemic manifestations were reported.

Following an inadequate response to conventional disease-modifying antirheumatic drug therapy, subcutaneous TCZ (Actemra®) was initiated approximately four months before her initial Oral Medicine visit at a dose of 162 mg/0.9 mL once weekly. Her medication history also included leflunomide (Arava®) 20 mg daily, amlodipine 5 mg twice daily, hydrochlorothiazide 25 mg daily, metformin (Glucophage®), and cetirizine (Artiz®) 10 mg as needed. The patient had previously been treated with denosumab (Prolia®) for osteoporosis, which had been discontinued seven months before initiation of TCZ therapy. A history of allergy to amoxicillin/clavulanate (Augmentin®) and other unspecified medications was documented.

The patient reported that the first oral ulcerative episode developed approximately 24 h after the first TCZ injection, with similar episodes recurring following subsequent weekly injections. At the initial Oral Medicine assessment, she reported that the lesions generally developed within the first day after TCZ administration, were usually one or two in number, increased in size, and caused pain with impairment of eating and speech. The duration varied between episodes; the clinical record documented some episodes lasting approximately 2–4 days, whereas other episodes during follow-up lasted longer.

During active episodes, the lesions were clinically characterized as shallow, painful, aphthous-like ulcerations predominantly affecting non-keratinized oral mucosa, particularly the labial and buccal mucosa (Figure 2). The soft palate and floor of the mouth were also involved during some episodes. The lesions had regular borders, a whitish fibrinous base, and a surrounding erythematous halo. No associated extraoral lesions, genital ulceration, or ocular involvement were identified. At one documented examination, no active ulceration was present, consistent with the recurrent and episodic nature of the condition.

**Figure 2:**
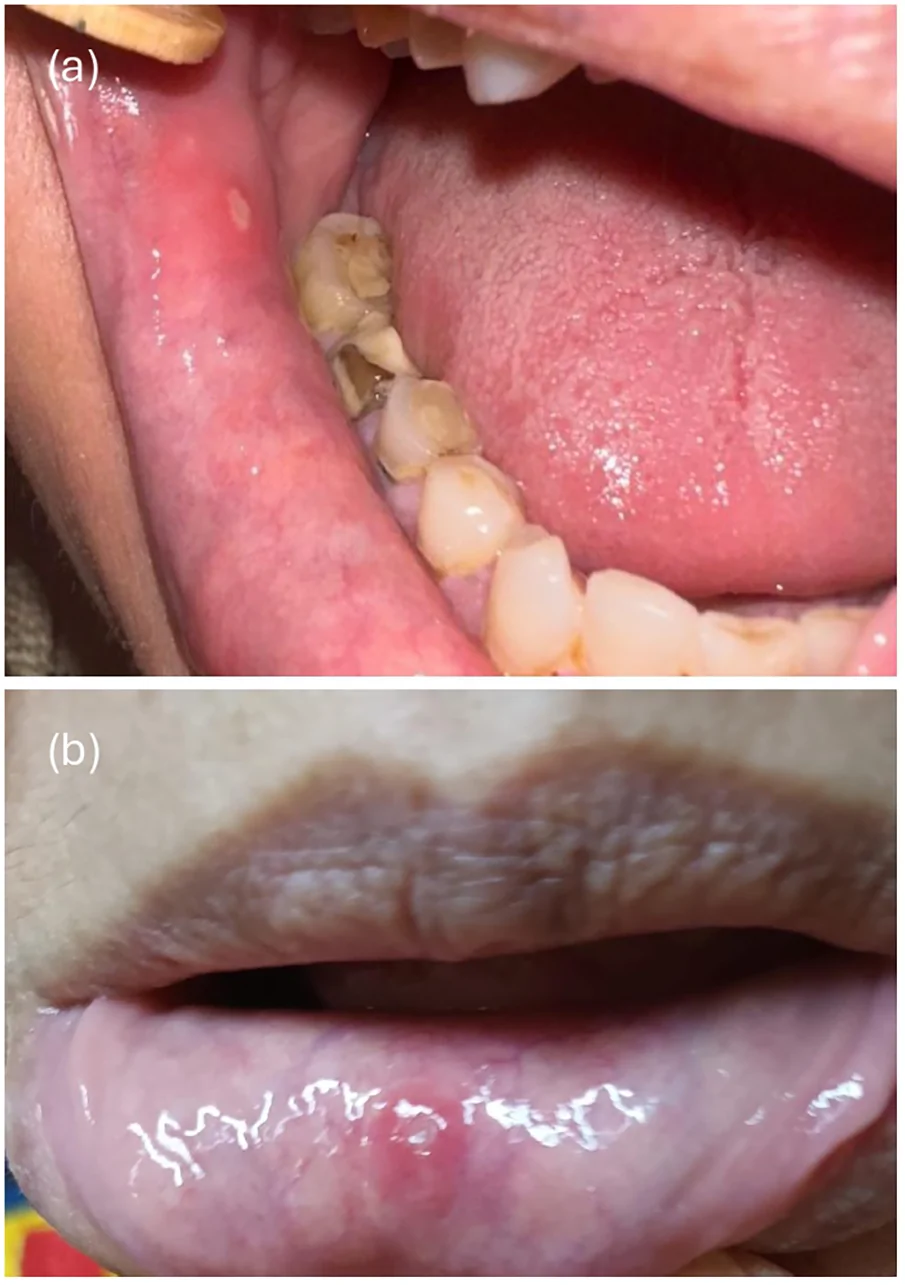
**(a)** Aphthous-like ulcer on the right labial commissure mucosa; **(b)** aphthous-like ulcer on the lower labial mucosa, both measuring approximately 3 mm in diameter with a fibrinous base and surrounding erythematous halo, appearing within 24 h of subcutaneous tocilizumab administration.

Oral examination also identified several potential local contributing factors, including generalized plaque and calculus accumulation, marginal gingivitis, multiple defective restorations, prepared teeth without definitive crowns, incomplete prosthodontic rehabilitation, and a broken crown with sharp edges in the mandibular left second molar region. No clinical evidence of hyposalivation was identified.

A diagnostic evaluation was undertaken to assess potential alternative causes of recurrent oral ulceration. Complete blood count, including the absolute neutrophil count, was within the reference range, with no clinically significant hematologic abnormalities. Serum iron, ferritin, vitamin B12, and folate were also within their respective reference ranges. Testing for herpes simplex virus (HSV) was negative. Biopsy was not considered clinically indicated because the lesions were recurrent rather than persistent and no suspicious lesion was present at the time of assessment.

Management was undertaken in coordination with the patient's rheumatologist and dental care team. Clobetasol propionate 0.05% (Dermovate®) was prescribed for topical application to affected mucosal sites twice daily for up to five days during active episodes. This provided partial symptomatic relief, although recurrent lesions, particularly at less accessible sites such as the soft palate, remained troublesome. Following consultation with the rheumatologist, colchicine 0.5 mg orally once daily was initiated. Chlorhexidine gluconate 0.12% mouthwash was prescribed at 10 mL twice daily for 30 s after meals. Because the defective restorations, sharp dental surfaces, plaque accumulation, gingival inflammation, and incomplete prosthodontic rehabilitation represented potential sources of local irritation, restorative and prosthodontic treatment and improvement in oral hygiene were also undertaken.

Following her initial Oral Medicine assessment, TCZ was continued, and the patient received a further 13 weekly doses during follow-up. Oral ulcerative episodes continued to recur during this period. Following the combined therapeutic and dental interventions, the patient reported a reduction in the severity and duration of the episodes. Pain intensity decreased from 9/10 to 3/10 on a numerical rating scale, although the episodes remained clinically significant and continued to affect her quality of life. Further improvement was observed following completion of prosthodontic rehabilitation.

TCZ was subsequently discontinued because of limited availability and replaced with certolizumab pegol (Cimzia®), administered subcutaneously at 400 mg at weeks 0, 2, and 4, followed by a maintenance dose of 200 mg every two weeks. Colchicine and topical oral treatments were discontinued when TCZ was withdrawn. During the subsequent six-month follow-up period, the oral ulcerative episodes progressively decreased in frequency and severity, with noticeable improvement after approximately three months. By six months, the episodes had become infrequent, occurring approximately once every eight weeks, and were milder and of shorter duration. At the updated follow-up, nine months after TCZ discontinuation, the patient reported complete resolution of the oral ulcerations, with no further episodes. Although the gradual improvement and eventual resolution are compatible with a treatment-related association, the concurrent withdrawal of TCZ, initiation of certolizumab, previous supportive therapies, and correction of local oral factors prevent attribution of the improvement to any single intervention.

Causality was assessed using the World Health Organization-Uppsala Monitoring Centre (WHO-UMC) system for standardized case causality assessment (30). The relationship between TCZ exposure and the oral ulcerations was classified as possible. This classification reflects the reasonable and repeatedly observed temporal relationship between weekly TCZ administration and ulcerative episodes, while acknowledging plausible alternative or contributory explanations, including local mechanical irritation, poor oral hygiene and gingival inflammation, concomitant medications, and the simultaneous introduction of several supportive and dental interventions. In addition, oral ulceration persisted, although at a lower frequency and severity, for six months after TCZ withdrawal, constituting an incomplete early dechallenge; complete resolution was documented only at the 9-month re-evaluation. The WHO-UMC classification therefore supports a possible association but does not establish that TCZ caused the lesions.

## Discussion

This study presents a case of recurrent oral ulcerations temporally associated with TCZ therapy, supported by a scoping review of the literature. Oral ulcerative events have been reported during TCZ exposure across diverse clinical settings and patient populations (7, 8, 10, 20, 26, 27). However, the available evidence is heterogeneous, and the relationship between TCZ exposure and oral ulceration remains incompletely established.

Evidence from clinical trials further indicates that oral ulcerative events have been reported during TCZ exposure; however, findings are heterogeneous and do not establish a consistent causal relationship. In the phase I study by Qian et al., oral ulcers occurred in 10/40 (25.0%) participants receiving the HS628 biosimilar and 9/37 (24.3%) receiving reference TCZ after a single 4 mg/kg intravenous infusion; importantly, both groups received an active IL-6 inhibitor and there was no untreated placebo group (20). Similarly, Schwabe et al. reported mouth ulceration in 23/685 (3.4%) healthy adults receiving a single subcutaneous dose of either TCZ or its biosimilar, with 1.9% of cases considered treatment-related (21). Among randomized trials in RA, SATORI reported stomatitis in 7/61 (11.5%) TCZ-treated patients compared with 0/64 controls (18), whereas SAMURAI reported stomatitis in 9/157 (5.7%) TCZ-treated patients compared with 13/145 (9.0%) receiving conventional DMARDs (17). In OPTION, mouth ulcers were reported in 2% of patients in each TCZ dose group compared with 0.5% in the placebo group (19), while TOWARD also documented mouth ulceration and stomatitis among treatment-emergent adverse events (4). Collectively, these variable findings indicate that oral ulcerative events can occur during TCZ treatment but are not uniformly increased compared with control groups, supporting a possible association rather than establishing causality.

The occurrence of oral ulceration in healthy volunteers is particularly informative because it minimizes confounding from underlying inflammatory disease (20, 21). Nevertheless, these phase I and biosimilarity studies were primarily designed to assess pharmacokinetics, bioequivalence, and general safety rather than to determine the incidence or causality of oral ulceration. In addition, the absence of an untreated comparator limits causal interpretation. These studies therefore demonstrate a temporal association between TCZ exposure and oral ulcerative events, but they do not establish that IL-6 inhibition directly caused the lesions. Their findings also should not be extrapolated to estimate the incidence of oral ulceration during long-term TCZ therapy. Overall, oral ulcerative events associated with TCZ remain incompletely characterized, and their true frequency, clinical phenotype, and causal relationship to treatment require further investigation.

In contrast, studies involving patients with systemic inflammatory conditions demonstrated more complex and clinically distinct patterns. In Behçet's disease, pre-existing oral ulceration sometimes persisted, showed limited therapeutic response, or worsened during TCZ treatment, rather than representing a newly developed adverse event (10, 26, 28). Similarly, Terreaux et al. described recurrence or persistence of pre-existing oral ulceration in a patient with MAGIC syndrome during TCZ therapy (9). By contrast, *de novo* treatment-emergent oral events were described by Tada et al., Samimi et al., and Vitiello et al.; notably, Samimi et al. reported resolution after TCZ withdrawal and recurrence following rechallenge, providing a stronger temporal relationship with treatment exposure (7, 8, 27). These findings emphasize the importance of distinguishing *de novo* treatment-emergent ulceration from persistence, lack of therapeutic efficacy, or paradoxical worsening of pre-existing disease-related mucocutaneous manifestations.

The findings of the present case are consistent with a temporal association between TCZ exposure and oral ulceration. The patient developed recurrent oral ulcerations shortly after initiation of TCZ, with lesions appearing within 24 h of administration and recurring following subsequent doses. This repeated temporal pattern is consistent with treatment-related association; however, it does not by itself establish causality. Alternative causes of oral ulceration, including infectious and systemic conditions, were considered during the clinical assessment. In the present case, the ulcerations were multiple, shallow, painful, and predominantly involved non-keratinized oral mucosa. However, morphology and anatomical distribution were inconsistently reported in the broader literature, precluding identification of a uniform clinical phenotype (7, 8, 10).

The underlying pathophysiology remains incompletely understood. IL-6 plays a central role in epithelial regeneration, maintenance of mucosal integrity, and immune regulation. Inhibition of IL-6 signaling may impair epithelial turnover and delay wound healing, thereby predisposing to mucosal breakdown. In addition, TCZ-associated neutropenia may further compromise host defense mechanisms, increasing susceptibility to mucosal injury (4–6). These proposed mechanisms remain speculative and cannot be confirmed from the available clinical evidence.

Comparison with other IL-6–targeted therapies provides additional perspective. Sarilumab, like TCZ, inhibits IL-6 signaling through blockade of IL-6R*α* and has demonstrated a broadly similar safety profile in comparative studies, particularly with respect to infections, neutropenia, and laboratory abnormalities (31). However, oral ulceration has not been consistently identified as a prominent adverse event in clinical trials. Siltuximab, which targets the IL-6 ligand rather than its receptor and is primarily used for multicentric Castleman disease, has likewise not established oral ulceration as a characteristic adverse event (32). Current evidence is therefore insufficient to determine whether oral ulceration represents a class effect of IL-6 pathway inhibition, a drug-specific phenomenon, or an event influenced by patient-related and local mucosal factors. Further comparative studies and pharmacovigilance analyses are warranted.

Management of TCZ-associated oral ulcerations remains largely empirical. In several previously reported cases, including those reported by Samimi et al. and Vitiello et al., TCZ was discontinued because of symptom severity, with subsequent clinical improvement after withdrawal (7, 8). In contrast, the present case demonstrates that continuation of TCZ may be feasible in selected patients when accompanied by appropriate supportive and dental care. TCZ was continued for a further 13 weekly doses during follow-up while topical clobetasol, chlorhexidine mouthwash, colchicine, optimization of oral hygiene, and restorative and prosthodontic treatment were introduced. During this period, the patient experienced a progressive reduction in the severity and duration of ulcerative episodes, with pain decreasing from 9/10 to 3/10. Further improvement was noted after completion of prosthodontic rehabilitation. However, because several therapeutic and dental interventions were introduced concurrently while TCZ was continued, the contribution of any single intervention to the observed improvement cannot be determined. Collectively, these findings support a multidisciplinary management approach addressing both systemic therapy and potentially contributory local oral factors, while suggesting that immediate TCZ discontinuation may not be necessary in every patient with treatment-associated oral ulceration.

Colchicine was one component of the multimodal supportive management used in this case. Its initiation was temporally followed by a reduction in the severity and duration of ulcerative episodes. Colchicine has anti-inflammatory effects, including inhibition of neutrophil chemotaxis, and is used in conditions characterized by recurrent oral ulceration, including recurrent aphthous stomatitis and Behçet's disease (33–35). A similar approach was reported by Samimi et al., in which colchicine was used during TCZ therapy (7). Nevertheless, because colchicine was introduced alongside other local and dental interventions, its individual therapeutic contribution cannot be established. Moreover, evidence from individual cases is insufficient to establish the efficacy of colchicine specifically for TCZ-associated oral ulceration or to confirm an underlying neutrophil-mediated mechanism.

The subsequent switch from TCZ to certolizumab pegol was associated with further improvement in ulcer frequency and severity. Although oral ulcerations persisted at a reduced frequency and severity during the first six months after TCZ withdrawal, complete resolution with no further episodes was documented at the updated 9-month re-evaluation. This delayed resolution represents an incomplete early dechallenge and limits attribution of improvement solely to TCZ withdrawal. Moreover, improvement had already begun before TCZ discontinuation, and several therapeutic and dental interventions were implemented concurrently. Accordingly, the eventual resolution cannot be attributed solely to withdrawal of TCZ or initiation of certolizumab pegol. Current evidence is insufficient to determine whether switching biologic therapy provides a specific benefit in TCZ-associated oral ulceration.

Beyond reports specifically addressing oral ulceration, systematic reviews provide broader context regarding the efficacy and safety of TCZ in inflammatory diseases. In rheumatoid arthritis, systematic reviews and meta-analyses have demonstrated the clinical efficacy of TCZ while also identifying recognized adverse events, particularly infections and laboratory abnormalities (36, 37). In giant cell arteritis, TCZ has also demonstrated therapeutic benefit in combination with glucocorticoid tapering regimens (38). In Behçet's disease, Akiyama et al. reported benefit for several systemic manifestations, although mucocutaneous responses were variable (11). Within this broader safety context, oral ulcerative events remain comparatively poorly characterized, and their frequency and causal relationship with TCZ have not been established.

This review has several limitations. The available evidence was heterogeneous and comprised clinical trials, observational studies, and case-report publications conducted in different patient populations and clinical settings. Reporting of oral-event morphology, anatomical distribution, timing of onset, duration, management, and resolution was inconsistent across studies, limiting direct comparison and precluding quantitative synthesis or reliable estimation of event frequency. In several studies, oral ulceration or stomatitis was reported only as an adverse-event term without detailed clinical characterization. The included populations also differed with respect to underlying disease, concomitant medications, TCZ regimen, and route of administration. In addition, some reports involved persistence or worsening of pre-existing disease-related ulceration rather than newly occurring treatment-emergent events. As expected for a scoping review, no formal causal inference or treatment-effect estimate can be made from the available evidence. In the presented case, although the close temporal relationship and recurrence following repeated TCZ administration are consistent with a treatment-related association, local factors including poor oral hygiene, mechanical irritation, and incomplete prosthodontic rehabilitation may also have contributed to lesion persistence or severity. Accordingly, the available evidence does not establish a definitive causal relationship between TCZ exposure and oral ulceration or permit reliable estimation of its frequency.

In conclusion, oral ulcerative events are infrequently reported and potentially underrecognized during TCZ therapy. The available evidence documents temporal associations in some cases, including recurrence following rechallenge, but remains insufficient to establish a definitive causal relationship. Pre-existing disease-related ulceration should be distinguished from newly occurring treatment-emergent events when evaluating patients receiving TCZ. Multidisciplinary assessment, attention to local oral factors, and exclusion of alternative causes may assist clinical management. Further studies are needed to better characterize the frequency, clinical phenotype, potential mechanisms, and optimal management of these events.
